# The biochemical aftermath of anti-amyloid immunotherapy

**DOI:** 10.1186/1750-1326-5-39

**Published:** 2010-10-07

**Authors:** Chera L Maarouf, Ian D Daugs, Tyler A Kokjohn, Walter M Kalback, R Lyle Patton, Dean C Luehrs, Eliezer Masliah, James AR Nicoll, Marwan N Sabbagh, Thomas G Beach, Eduardo M Castaño, Alex E Roher

**Affiliations:** 1 The Longtine Center for Neurodegenerative Biochemistry, Banner Sun Health Research Institute, Sun City, AZ, USA; 2 Department of Microbiology, Midwestern University, Glendale, AZ, USA; 3 Department of Neurosciences, University of California San Diego, La Jolla, CA, USA; 4 Division of Clinical Neurosciences, University of Southampton School of Medicine, Southampton, UK; 5 Cleo Roberts Center for Clinical Research, Banner Sun Health Research Institute, Sun City, AZ, USA; 6 Department of Neuropathology, Banner Sun Health Research Institute, Sun City, AZ, USA; 7 Fundacion Instituto Leloir, Buenos Aires, Argentina

## Abstract

**Background:**

Active and passive immunotherapy in both amyloid-beta precursor protein (APP) transgenic mice and Alzheimer's Disease (AD) patients have resulted in remarkable reductions in amyloid plaque accumulation, although the degree of amyloid regression has been highly variable. Nine individuals with a clinical diagnosis of AD dementia were actively immunized with the Aβ peptide 1-42 (AN-1792) and subjected to detailed postmortem biochemical analyses. These patients were compared to 6 non-immunized AD cases and 5 non-demented control (NDC) cases.

**Results:**

All patients were assessed for the presence of AD pathology including amyloid plaques, neurofibrillary tangles and vascular amyloidosis. This effort revealed that two immunotherapy recipients had dementia as a consequence of diseases other than AD. Direct neuropathological examination consistently demonstrated small to extensive areas in which amyloid plaques apparently were disrupted. Characterization of Aβ species remnants by ELISA suggested that total Aβ levels may have been reduced, although because the amounts of Aβ peptides among treated individuals were extremely variable, those data must be regarded as tentative. Chromatographic analysis and Western blots revealed abundant dimeric Aβ peptides. SELDI-TOF mass spectrometry demonstrated a substantive number of Aβ-related peptides, some of them with elongated C-terminal sequences. Pro-inflammatory TNF-α levels were significantly increased in the gray matter of immunized AD cases compared to the NDC and non-immunized AD groups.

**Conclusions:**

Immunotherapy responses were characterized by extreme variability. Considering the broad range of biological variation that characterizes aging and complicates the recognition of reliable AD biomarkers, such disparities will make the interpretation of outcomes derived from epidemiologic and therapeutic investigations challenging. Although in some cases the apparent removal of amyloid plaques by AN-1792 was impressive, proportionate alterations in the clinical progression of AD were not evident. The fact that plaque elimination did not alter the trajectory of decline into dementia suggests the likelihood that these deposits alone are not the underlying cause of dementia.

## Background

Alzheimer's disease (AD) dementia affects over 26 million elderly individuals worldwide with this number projected to quadruple by 2050 [1]. In the USA alone, 5.3 million people are afflicted with AD at an estimated annual cost of $172 billion [2]. Given these alarming epidemiological data, devising strategies and therapeutic interventions to prevent, mitigate or delay the age of onset of this dementia is urgent.

The deposition of amyloid-beta (Aβ) peptides in the brains of patients with AD has been a principal focus of intense research since the seminal publications of Glenner and Wong [3] and Masters et al. [4]. The observation of profuse accumulation of parenchymal and vascular Aβ peptides in AD brains was integrated into the amyloid cascade hypothesis as the central causative factor in the pathogenesis of AD dementia [5,6]. Genetic and biochemical studies of amyloid-beta precursor protein (APP), presenilin (PS) 1 and PS2 mutations, all of which enhance amyloid deposition, strongly support this hypothesis. For many AD researchers, the amyloid hypothesis has attained a status of virtual dogma. However, there are dissidents who have critically questioned this powerful tenet [7,8].

The APP and Aβ peptides are evolutionarily conserved molecules with multiple functions. It has been suggested that Aβ may serve a neurogenic function in the development of neural stem cells [9]. It has also been postulated that Aβ binds neurotoxic substances and that amyloidosis stimulates their phagocytic removal, thus representing a physiological response to injury [10,11]. A decline in β- and γ-secretase activities decreases Aβ production, inducing neuronal death [12]. The Aβ peptides are powerful modulators of microglial activation [13,14], have vasoconstrictor activity [15] that may have a protective role in neuroinflammation and inhibit angiogenesis [16]. A hemostatic function for the Aβ deposited in the walls of the cerebral microvasculature has also been postulated [17,18].

Transgenic (Tg) mouse models have been engineered to express well-characterized APP, PS and tau mutations. These mice are widely utilized to test the efficacy of various compounds and strategies intended to alleviate the deleterious effects of Aβ peptide accumulation or promote its specific clearance. Special attention has been devoted to amyloid peptide immunization therapies to determine their effects on pathology and cognition. Both active and passive immunotherapy in Tg mice successfully reduced amyloid plaque accumulation and cognition deficits (reviewed in reference [19]). Unfortunately, these promising interventions have yet to produce unequivocal therapeutic benefit in human clinical trials. Several factors may explain discrepancies between Tg animal and human AD patient responses to amyloid immunotherapy. Transcriptome analyses have revealed that the murine response to aging is very different from that of humans [20]. In addition, differences in lifespan, intrinsic rates of aging, the relative simplicity of mouse brains and the nature of the induced lesions artificially imposed upon the mice models, may also account for these differences [21-23].

Multiple clinical and pathological observations, in demented individuals, including the biochemical composition of amyloid plaques, suggest that the pathogenesis of AD is a complex and multifactorial process involving more than Aβ. In support of these tenets are the neuropathological, biochemical and immunochemical studies performed on the brains of Aβ-immunized individuals. Although there were obvious extensive areas in which amyloid plaques were absent, the amount of vascular amyloid and soluble Aβ apparently increased while neurofibrillary tangles (NFT) were unaffected [24-31]. More importantly, despite anti-Aβ immunization during a period of mild to moderate cognitive impairment, all patients exhibited continued, progressive dementia and eventually died with AD [29]. Furthermore, Aβ immunotherapy clinical trials have been fraught with frustrations. The AN-1792 clinical trial was halted after 6% of the immunized population developed aseptic meningoencephalitis [32]. More recently, the Bapineuzumab passive immunotherapy clinical trial was complicated by the development of brain vasogenic edema in some individuals carrying the apolipoprotein (ApoE) *ε4 *allele, which compelled the elimination of these subjects from the trial [33]. Notwithstanding these adverse events, recent analyses of AD AN-1792 immunized individuals revealed an apparent reduction in neurite and tau pathology [34,35].

In this paper, we report the neuropathological and biochemical changes observed in a cohort of 9 individuals clinically diagnosed with AD that were actively immunized with fibrillar Aβ1-42 under the label of AN-1792. We compare the present results with our previous investigations of 2 AN-1792 immunized cases [28], and with data derived from 6 untreated AD cases and 5 non-demented control (NDC) individuals.

## Methods

### Human Subjects

The study involved 9 individuals clinically diagnosed with AD who were actively immunized with the Aβ peptide 1-42 (AN-1792). Six of these cases were provided by Dr. J. Nicoll, University of Southampton School of Medicine (USSM) and 3 by Dr. E. Masliah, University of California San Diego (UCSD) (Table 1). For comparison, 6 non-immunized AD cases and 5 NDC cases were provided by Dr. T. Beach, Banner Sun Health Research Institute (BSHRI). In the immunized group (n = 9), the mean patient age of death was 81 years, 2 were female and 7 were male with a mean disease duration of 8 years. In the NDC individuals, cases # 1-5, the mean age of death was 82 years (2 females and 3 males), and in the AD group, cases # 6-11, the mean age of death was 82 years (5 females and 1 male) with a mean disease duration of 9 years. Their individual ages, gender, ApoE genotype, disease duration, and when applicable, immunogen doses, number of injections and antibody titers as well as the survival time after the application of the first immunization doses are given in Table 1. Of the 9 immunized individuals with a clinical diagnosis of AD, upon postmortem examination, two cases were classified as non-AD dementia cases (# 21 from USSM and # 22 from UCSD), having the neuropathological diagnoses of progressive supranuclear palsy (PSP) and hippocampal sclerosis (HS) with severe frontal lobe atrophy, respectively. One case (# 19) was neuropathologically diagnosed as a Lewy body variant of AD.

**Table 1:** Clinical characteristics and immunization details of study subjects

Case ID	Expired age (years)	Gender	ApoE genotype	Disease Duration (years)	Imm. Dose (μg)	# of injections	Mean antibody response (ELISA units)	PIST (months)
**NDC**								
1-BSHRI	77	F	2/3	-	-	-	-	-
2-BSHRI	81	F	4/4	-	-	-	-	-
3-BSHRI	83	M	3/4	-	-	-	-	-
4-BSHRI	88	M	2/3	-	-	-	-	-
5-BSHRI	81	M	3/3	-	-	-	-	-
**Mean**	**82**							
								
**AD**								
6-BSHRI	76	F	4/4	6	-	-	-	-
7-BSHRI	82	M	3/4	7	-	-	-	-
8-BSHRI	86	F	3/4	5	-	-	-	-
9-BSHRI	80	F	3/3	12	-	-	-	-
10-BSHRI	85	F	3/3	9	-	-	-	-
11-BSHRI	81	F	4/4	15	-	-	-	-
**Mean**	**82**			**9**				
								
Immunized AD*								
12-USSM	71	F	3/4	10	225	8	1:4072	44
13-USSM	81	M	n/a	7	50	8	1:1707	57
14-USSM	82	M	3/4	6	50	8	1:4374	60
15-USSM	81	M	4/4	11	225	7	1:491	63
16-USSM	88	F	3/3	11	50	7	1:137	86
19-UCSD	78	M	3/4	5	50	1	n/a	36
20-UCSD	86	M	3/4	9	50	1	n/a	60
**Mean**	**81**			**8**		**6**		**58**
								
Immunized non-ADD*								
21-USSM PSP	79	M	2/3	6	50	8	< 1:100	51
22-UCSD HS	80	M	3/3	5	50	1	n/a	48

NDC, non-demented control; AD, Alzheimer's disease; non-ADD, non-Alzheimer's disease dementias; ApoE, apolipoprotein E; Imm., immunization; PIST, post-immunization survival time; F, female; M, male; BSHRI, Banner Sun Health Research Institute; USSM, University of Southampton School of Medicine; USCD, University of California San Diego; n/a, not available; PSP, progressive supranuclear palsy; HS, hippocampal sclerosis.

* Immunization data for USSM cases taken from Holmes, et al [29], Boche, et al [30] and Boche, et al [35].

### Morphological assessments

The BSHRI AD and NDC cases were assessed for total plaque score, plaque density, total NFT score, Consortium to Establish a Registry for Alzheimer's disease (CERAD), neuritic plaque score, Braak stage, total WMR and total CAA score (see Table 2). Detailed evaluations of these neuropathological parameters are given in a previous publication [36]. Semi-quantitative appraisal of plaque clearance, subjectively attributed to immunotherapy, was estimated as none (0), mild (+), moderate (++) and extensive (+++). The evaluation of CAA content in the UCSD cases was performed by lysing 10 cubes of cerebral cortex tissue (~ 1 cm per side) in 100 ml 10% SDS, 10 mM Tris-HCl pH 7.4. After 48 h of continuous stirring at room temperature the only remaining structures were the tufts of insoluble blood vessels and attached amyloid deposits. After removal of the detergent, the vessels were air dried on glass slides and stained with thioflavine-S [17].

**Table 2:** Neuropathology of study subjects

Case ID	Aβ42 load (%)	Plaque clear-ance	Brain weight (g)	Total WMR score	Total plaque score	Plaque density	Total NFT score	Cerad NP	Braak stage	Total CAA score
**NDC**										
1	-	-	950	n/a	6.0	zero	2.0	not AD	I	n/a
2	-	-	1275	0	7.6	sparse	3.0	not AD	II	2
3	-	-	1385	0	1.0	zero	1.0	not AD	I	0
4	-	-	1260	4	6.8	moderate	4.3	possible AD	III	3
5	-	-	1190	0	12.5	sparse	6.4	not AD	III	5
										
**AD**										
6	-	-	1100	0	13.5	moderate	8.8	probable AD	IV	4
7	-	-	1145	0	13	frequent	13	definite AD	V	5
8	-	-	1055	n/a	13.25	frequent	8.75	definite AD	V	4
9	-	-	765	9	12.5	frequent	14.5	definite AD	VI	1
10	-	-	960	0	12.25	frequent	12	definite AD	V	0
11	-	-	970	10	13.25	frequent	15	definite AD	VI	10
										
Immunized AD*										
12	4.68	+	n/a	n/a	n/a	n/a	n/a	n/a	VI	n/a
13	3.32	++	1120	n/a	n/a	n/a	n/a	n/a	VI	n/a
14	0.05	+++	1200	n/a	n/a	n/a	n/a	n/a	VI	n/a
15	2.71	++	n/a	n/a	n/a	n/a	n/a	n/a	VI	n/a
16	2.99	+	n/a	n/a	n/a	n/a	n/a	n/a	VI	n/a
19	n/a	n/a	1208	n/a	n/a	n/a	n/a	n/a	III	n/a
20	n/a	n/a	1162	n/a	n/a	n/a	n/a	n/a	IV	n/a
										
Immunized non-ADD*										
21 PSP	0.75	0	n/a	n/a	n/a	n/a	n/a	n/a	n/a	n/a
22 HS	n/a	n/a	1280	n/a	n/a	n/a	n/a	n/a	n/a	n/a

NDC, non-demented controls; AD, Alzheimer's disease; non-ADD, non-Alzheimer's disease dementias; PSP, progressive supranuclear palsy; HS, hippocampal sclerosis; WMR, white matter rarefaction; NFT, neurofibrillary tangle; NP, neuritic plaque; CAA, cerebral amyloid angiopathy; n/a, not available. Plaque clearance designations correspond to 0, none; +, little evidence or active process; ++, moderate/patchy; +++, complete.

*Aβ42 load and Braak stage for USSM cases taken from Holmes, et al [29], Boche, et al [30] and Boche, et al [35].

Clinical, neuropathological and long-term effects of Aβ42 immunization for the AD patients # 12-15, originating from USSM, have been thoroughly described elsewhere [29,30,35]. The neuropathology reports for cases # 19, 20 and 22 from UCSD are summarized in Table 3.

**Table 3:** Summary of Clinico-Neuropathology Analysis in UCSD AN-1792-Immunized Cases

Case ID	**Blessed **[63]	MMSE	Total plaque	Neuritic plaques	Tangles	CAA	Neuropathology diagnosis
19	26	8	23	7	0	3	Lewy body Variant of AD
20	17	18	50	0	0	2	AD
22	28	10	4	3	0	0	HS, severe frontal lobe atrophy

MMSE, mini-mental state examination; CAA, cerebral amyloid angiopathy; AD, Alzheimer's diease; HS, hippocampal sclerosis. The counts are representative from the frontal cortex. For each case, 3 sections were analyzed. Total plaques include diffuse and mature with a maximum score of 50 (100 × magnification). Neuritic plaques are defined as those containing a corona of dystrophic neurites with a maximum score of 50 (100 × magnification). Tangles were assessed at 400 × magnification with a maximum score of 50. CAA was assessed as 0, none; 1, mild; 2, moderate; 3, severe using thioflavine-S staining.

### Quantification of soluble and insoluble Aβ by ELISA

All steps were performed at 4°C. Gray matter tissue (100 mg) was homogenized with a Teflon tissue grinder in 6 volumes of 20 mM Tris-HCl, 5 mM EDTA, pH 7.8 with protease inhibitor cocktail (PIC, Roche Diagnostics, Mannheim, Germany). The homogenates were centrifuged at 435,000 × *g *for 20 min in an Optima TLA-ultracentrifuge using a 120.2 rotor (Beckman, Fullerton, CA). The supernatant was saved as the soluble Aβ fraction and total protein quantified with the Pierce BCA protein assay (Rockford, IL). Four-hundred mg each of gray and white matter tissue was homogenized in 3 ml of 90% glass distilled formic acid (GDFA) and centrifuged at 250,000 × *g *for 20 min at 4°C in an Beckman LE-80K ultracentrifuge using a SW41 rotor (Beckman). The supernatant was collected carefully avoiding the top fat layer. Specimens were homogenized in 90% GDFA with the aim of fully solubilizing all forms of Aβ (fibrillar, diffuse, membrane-bound, and intra- and extra-cellular oligomeric species). High-speed centrifugation permitted the removal of all lipids including membrane-associated forms which are totally disrupted by the GDFA and form a compact aggregate at the top of the centrifuge tube. The entire volume from each case was submitted to fast protein liquid chromatography (FPLC) size-exclusion Superose-12 chromatography in 80% GDFA mobile phase (see below). The Aβ peptide-containing fractions were collected and pooled from each run and reduced to 2 ml by vacuum centrifugation (SpeedVac; Savant/Thermo, Waltham, MA). To remove the acid, each case was dialyzed in 1000 MW cutoff tubing against 2 changes of water (1 h each) and 2 changes of 0.1 M ammonium bicarbonate solution (1 h each). Samples were freeze-dried and lyophilized. The samples were then reconstituted in 500 μl of 5 M guanidine hydrochloride (GHCl) prepared in 50 mM Tris-HCl, pH 8.0 and shaken overnight at 4°C. Total protein was quantified by the Pierce BCA protein assay. The ELISA kits to quantify Aβ40 and Aβ42 were obtained from Invitrogen (Carlsbad, CA) and Innogenetics (Gent, Belgium), respectively, and performed following the manufacturer's instructions.

### Quantification of tumor necrosis factor-α (TNF-α) by ELISA

All steps were performed at 4°C and followed a previously published protocol [37]. Gray matter (100 mg) was homogenized in 20 volumes of 20 mM HEPES, 1.5 mM EDTA, pH 7.4 in PIC (Roche) with an Omni TH electric tissue grinder and the samples centrifuged at 3000 × *g *for 15 min in an IEC Centra CL3R centrifuge (Thermo, Waltham, MA). The supernatant was then collected and centrifuged at 40,000 × *g *for 1 h with a 50.4 Ti rotor (Beckman) and Optima LE-80K ultracentrifuge (Beckman). The supernatant was again collected and total protein determined with a BCA protein assay (Pierce). Human TNF-α levels were measured with a kit from PromoKine (Heidelberg, Germany) according to the manufacturer's instructions.

### Western Blot Analysis

Western blots were performed as previously described [38]. Briefly, gray matter was homogenized in RIPA buffer (Sigma, St. Louis, MO), containing PIC (Roche). The homogenates were centrifuged, the supernatants collected and total protein quantified with a BCA protein assay kit (Pierce). The samples (25 μg of total protein loaded per lane) were separated by SDS-PAGE and then electrophoretically transferred onto 0.45 μm pore nitrocellulose membranes (Invitrogen), blocked with 5% non-fat milk in phosphate-buffered saline (PBS), 0.5% Tween 20 (Fluka, St. Louis, MO). The primary antibodies used in the experiments included 22C11 (recognizes amino acids 66-81 of APP; Millipore, Billerica, MA), CT9APP (recognizes the last 9 C-terminal amino acids of APP; Millipore) and anti-tau HT7 (recognizes amino acids 159-163; Pierce). The secondary antibodies used were either goat anti-mouse IgG conjugated horseradish peroxidase (HRP; 22C11 and anti-tau) or goat anti-rabbit IgG conjugated HRP (CT9APP) from Pierce. Protein signals were detected with SuperSignal WestPico Chemiluminescent (Pierce) substrate, CL-Xpose film (Pierce) and Kodak GBX developer and fixer (Sigma). Analysis was performed with a GS-800 calibrated densitometer (Bio-Rad, Hercules, CA) and Quantity One software (Bio-Rad). All membranes were stripped with Restore™ Western Blot Stripping Buffer (Pierce), washed in PBS and then re-blocked. Anti-mouse or anti-rabbit actin antibodies (Abcam, Cambridge, MA) were used to re-probe the blots for total protein normalization.

### Fast protein liquid chromatography

Cerebral cortex (~ 3 g) was minced and homogenized in 18 ml of 90% GDFA, allowed to stand at room temperature for 15 min and centrifuged at 240,000 × *g *for 1 h at 4°C. The separated lipid layer collected at the top of the tube was carefully removed and the small pellet at the bottom of the tube discarded. The intermediate supernatant was divided into 500 μl portions and frozen at -80°C. Each aliquot was submitted to size-exclusion FPLC using a Superose 12 column (10 × 300 mm, General Electric, Uppsala, Sweden), equilibrated with 80% GDFA. The chromatography was developed using 80% GDFA at a flow rate of 15 ml per h at room temperature and monitored at 280 nm. The fraction that eluted between 50-66 minutes, containing 2-8 kDa M_r _molecules, was collected and reduced to ~50 μl by vacuum centrifugation (Savant Instruments Inc), and stored at -80°C.

### High performance liquid chromatography (HPLC)

The 2-8 kDa FPLC fractions were separated by reverse-phase HPLC using a C8 column (4.6 × 250 mm, Zorbax SB, Mac Mod) using a linear gradient from 0-60% water-acetonitrile concentration containing 0.1% trifluoroacetic acid (TFA), developed at a flow rate of 1 ml per min over 120 min at 80°C. Absorbance was monitored at 214 nm and a total of 9 fractions collected and reduced in volume by vacuum centrifugation. To eliminate the acid, the specimens were washed with three changes of water (200 μl each) and the volume reduced by vacuum centrifugation. After the last wash, the volume was reduced and the samples re-solubilized in 2xLDS sample loading buffer (Invitrogen) with 50 mM dithiothreitol. Western blots were conducted as described above with anti-Aβ40 and anti-Aβ42 (Invitrogen) and CT9APP (Millipore) as primary antibodies and goat α-rabbit IgG HRP as the secondary antibody.

### Surface enhanced laser desorption/ionization-time of flight mass spectrometry (SELDI-TOF) mass spectrometry (MS). Aβ40/42 Method

HPLC peaks that were analyzed using Western blots and found to contain Aβ were subjected to SELDI-TOF MS following a previously published protocol [39]. Briefly, the capture antibodies, anti-Aβ40 and anti-Aβ42 antibodies (Invitrogen), were loaded onto PS20 ProteinChip arrays (Bio-Rad, Hercules, CA) at a concentration of 0.38 mg/ml. Either the HPLC Aβ-containing peaks or Aβ1-40 or 1-42 peptide standards (positive controls) were then applied. The molecular mass assignments resulted from 100 averaged shots in a Bio-Rad SELDI Protein Biology System II with external calibration attained using the ProteinChip Peptide Mass Calibration Kit (Bio-Rad).

### SELDI-TOF MS, 6E10 Method, ProteinChip β-Amyloid MPD Kit

The HPLC peaks containing Aβ were pooled together to yield one combined sample per case. All steps were performed at room temperature. The internal standard and calibrants were prepared according to the manufacturer's instructions (Bio-Rad). To each HPLC and calibrant sample (Aβ peptides: 1-16, 1-38, 1-40 and 1-42), 50 μl/ml of the internal standard AβCys1-24 (M_r _= 2,979.3) was added. Each spot on the ProteinChip arrays was equilibrated with 5 μl of PBS for 5 min, then loaded with 5 μl of sample or calibrant and incubated in a humidified chamber for 1 h. The samples/calibrants were removed and each array was washed 3 times in a 15 ml conical tube with 10 ml of wash buffer (PBS, 0.5% Triton X-100) for 5 min each then 3 times in PBS for 5 min each. To desalt the arrays, each chip was washed in 10 ml of 0.1 M HEPES for 5 min and then air dried. To 5 mg of α-cyano-4-hydroxycinnamic acid (CHCA), 200 μl of acetonitrile and 200 μl of 1.0% TFA were added. The solution was vortexed for 2 min then centrifuged at 1000 × *g *for 1 min to remove particulates. A 20% solution of CHCA was made from the saturated CHCA (diluted in a 1:1 ratio of acetonitrile and 1.0% TFA) and vortexed for 1 min. The 20% CHCA solution was applied (1 μl) to each spot and air dried. The molecular mass assignments and calibration was performed as described above.

## Results

### I. Clinical and Neuropathological Observations

**Figure 1:**
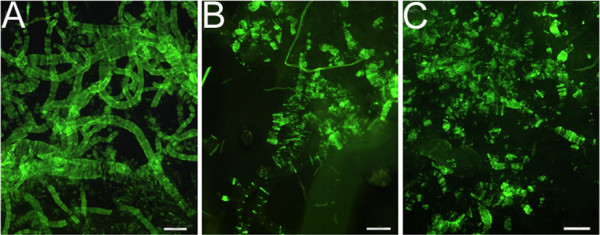
**Thioflavin-S of blood vessels to assess CAA in UCSD cases using SDS lysates of cerebral cortex**. A) Case # 19 demonstrated severe CAA. B) Case # 20 showed moderate CAA. C) Case # 22 exhibits moderate CAA, although this case was neuropathologically reported as having no CAA (Table 3). These differences may be due to different methodological assessments or sampling sites. Scale bar = 250 μm.

We examined 9 individuals clinically diagnosed with AD who were treated with the AN-1792 immunotherapy. Whole mount preparations of cortical blood vessels of cases # 19, 20 and 22, after removal of brain parenchyma by SDS, revealed the presence of insoluble CAA (Figure 1). For comparative reference, Table 2 shows the neuropathological parameters observed in 5 NDC individuals (cases # 1-5) and 6 non-immunized AD patients (cases # 6-11). The neuropathological changes observed in 5 immunized cases originating from USSM (cases # 12 -16), who were treated with the AN-1792 antigen, were described in previous publications [29,30,35]. Their Aβ42 load and semi-quantitatively estimated degree of plaque clearance and Braak stage are given in Table 2. This group of individuals each received 7-8 antigen injections, at 50 or 225 μg per dose, and had an average survival time of 62 months (range 44-86 months) after the initial immunization (Table 1). Four of these individuals were ApoE genotyped, and of these, three were carriers of the *ApoE ε4 *allele.

The USSM case # 21, clinically diagnosed as having AD, received 6 immunizations of 50 μg each (Table 1). However, on neuropathological examination, this case was reclassified as PSP. Accordingly, the Aβ42 load was very low (0.75%) (Table 2), and the levels of soluble and insoluble Aβ as measured by ELISA were also negligible (Table 4).

**Table 4:** ELISA quantification of TNF-α, Aβ40 and Aβ42

	GM	GM GDFA/GHCl-soluble Aβ			WM GDFA/GHCl-soluble Aβ			GM Tris-soluble Aββ		
	TNF-α pg/mg total protein*	**Aβ40 ng/mg total protein**	Aβ42 ng/mg total protein**#	Total Aβ ng/mg total protein***	**Aβ40 pg/mg total protein**	**Aβ42 pg/mg total protein**	**Total Aβ pg/mg total protein**	**Aβ40 pg/mg total protein**	**Aβ42 pg/mg total protein**	**Total Aβ pg/mg total protein**

**NDC**										
1	24	15	488	503	102	454	556	0	0	0
2	13	82	911	993	82	428	510	166	438	604
3	14	4	105	109	83	429	512	0	0	0
4	12	56	920	976	66	570	636	0	0	0
5	17	167	2168	2335	81	2596	2677	0	142	142
**Mean**	**16**	**65**	**918**	**983**	**87**	**836**	**923**	**28**	**97**	**124**

										
**AD**										
6	11	5405	8152	13557	101	4122	4223	116	138	254
7	13	374	5910	6284	923	2379	3302	0	88	88
8	13	179	4773	4952	90	5156	5246	148	93	241
9	13	394	2664	3058	136	965	1101	254	66	320
10	15	96	5344	5440	91	1931	2022	0	35	35
11	18	7169	2847	10016	8940	13669	22609	151	0	151
**Mean**	**14**	**2269**	**4948**	**7218**	**1714**	**4704**	**6417**	**112**	**70**	**182**

										
**Immunized AD**										
12	45	6347	58	6405	-	-	-	1260	0	1260
13	37	9925	1226	11151	-	-	-	13169	262	13431
14	32	252	31	283	-	-	-	0	0	0
15	32	1067	421	1488	-	-	-	629	26	655
16	43	8568	1570	10138	-	-	-	7556	142	7698
19	33	764	1565	2329	661	2141	2802	498	0	498
20	34	6315	7696	14011	7363	29669	37032	565	160	725
**Mean**	**34**	**4748**	**1795**	**6544**	**4012**	**15905**	**19917**	**3382**	**84**	**3467**

										
**Immunized non-ADD**										
21 PSP	30	1	18	19	-	-	-	0	0	0
22 HS	13	3	333	336	158	1245	1403	0	0	0

* p < 0.0001; **p = 0.026; ***p = 0.007 unpaired, 2-tailed t-tests between AD and NDC cases. # p = 0.039 unpaired, 2-tailed t-tests between non-immunized and immunized AD individuals. NDC, non-demented control; AD, Alzheimer's disease; non-ADD, non-Alzheimer's disease dementias; TNF, tumor necrosis factor; GM, gray matter; WM, white matter; GHCl, guanidine hydrochloride, GDFA, glass-distilled formic acid; PSP, progressive supranuclear palsy; HS, hippocampal sclerosis.

Table 3 gives an account of clinical and neuropathological data for the three cases provided by UCSD. Cases # 19 and # 20 were recognized after postmortem neuropathological examination as Lewy body variant of AD and AD, respectively. The immunized patient # 22, clinically diagnosed as AD, was found to be a case of HS on neuropathological examination and also had low levels of Aβ compared to the average of the immunized AD group (Table 4). All three individuals received only a single dose of the AN-1792 antigen.

### II. Aβ ELISA Quantification

Pivotal in the assessment of immunotherapy effectiveness is the quantification of Aβ40, Aβ42 and total Aβ peptides by ELISA, as Tris-soluble and GDFA/GHCl-soluble forms in gray matter (GM) and white matter (WM). Frozen WM was not available from USSM for ELISA analysis. These values are shown in Table 4. For comparison, analogous values observed in non-immunized AD and NDC individuals are also presented in Table 4. Significant differences in Aβ levels between the NDC and AD populations in both GM and WM tissue compartments were observed for GM GDFA/GHCl Aβ42 and total Aβ GM GDFA/GHCl levels (p = 0.0026 and 0.0066, respectively, unpaired, 2-tailed t-test).

The overall degree of variability in the total Aβ levels in the GM of the immunized AD cases is enormous, ranging from 283 to 14,011 ng/mg total protein (mean = 6,544) for GDFA/GHCl-soluble species and from 0 to 13,431 pg/mg of total protein (mean = 3,467) for the corresponding Tris-soluble Aβ fractions (Table 4). There are remarkable differences concerning the final outcome of immunotherapy as illustrated by subjects # 13 and 14. Both individuals received 8 AN-1792 injections of 50 μg (Table 1). In the latter case, the histological observations [29,30] were matched by ELISA quantification which revealed comparatively lesser amounts of total soluble and insoluble Aβ peptides, while case # 13 had the second highest levels of GDFA/GHCl-soluble Aβ and the highest levels of Tris-soluble Aβ observed. In contrast, case # 14 had the lowest levels of Aβ in both solvents compared to the immunized group. In these two individuals, the survival time since the first immunization was almost identical: 57 and 60 months, illustrating that the presence or absence of Aβ amyloid plaque pathology was apparently irrelevant for the progression and fatal outcome with AD dementia.

A comparison of the average Aβ levels between immunized and non-immunized AD groups also demonstrates large overall differences in soluble and insoluble Aβ content (Table 4). There is a statistically significant difference in the GDFA/GHCl-soluble Aβ42 between immunized and non-immunized individuals (p = 0.039), being lower in the former group. On the other hand, the levels of Aβ40 were increased paradoxically in the immunized cohort, almost to twice the mean quantities observed in the non-immunized AD control group, although the difference did not reach statistical significance. In one immunized individual (case # 14) the level of total Tris-soluble Aβ fell below the limit of detection. The mean of the remaining 6 immunized individuals was 22 times greater than the mean observed in the non-immunized AD cases.

Given the high degree of pathological variability encountered among NDC, AD and Aβ-immunized AD patients, as shown in Table 4, it is also important to consider weighing the data individually, rather than exclusively working with group mean values. For example, in the NDC group, both GM and WM GDFA/GHCl-soluble Aβ levels of case # 5 were abnormally high for this group and consequently skews the average values for this group upward to a range of 4-5-fold greater than the mean of the other members of the NDC group. Hence, this case should be classified as a non-demented 'high pathology control'. In the AD group, the GDFA/GHCl-soluble Aβ in WM of individual # 11 is abnormally high for this group and again skews the average values upward, being 7 times greater than the mean value observed in the rest of the group. In the immunized group, the WM GDFA/GHCl-soluble total Aβ level in case # 20 is 13 times greater than the values observed for case # 19. In the Tris-soluble Aβ fraction, the immunized AD individuals # 13 and # 16 have 21- and 12-fold more Aβ, respectively, than the remaining 5 individuals in this group. These elevated values are also reflected, although to a lesser degree, in the GM GDFA/GHCl-soluble Aβ fractions and are correlated with only moderate and mild plaque clearance [29,30]. Dixon's Q test [40] also identified these individuals as outliers.

Interestingly, 3 of the 7 immunized neuropathologically confirmed AD cases had a total GM GDFA/GHCl-soluble Aβ values substantially lower than the mean of the 6 non-immunized AD cases (7218 ng/mg total protein), suggesting that the antibodies may have removed Aβ in these cases. The remaining 4 immunized cases had values equal to or higher than the mean observed in the non-immunized AD group.

As mentioned above, of the 3 cases treated with the AN-1792 immunogen that were provided by UCSD, one was neuropathologically diagnosed as HS. The remaining 2 individuals, cases # 19 and # 20, exhibited the neuropathology of AD, as shown in Table 3. From the Aβ immunoassay standpoint, case # 19 had relatively moderate levels of GM and WM GDFA/GHCl-soluble Aβ peptides as well as Tris-soluble Aβ. In contrast, case # 20 showed the highest levels of GM and WM GDFA/GHCl-soluble Aβ peptides of the 20 cases under investigation including the 5 NDC and 6 non-immunized AD cases that served as controls.

In addition, there was also a high degree of variability in antibody response (Table 1). For example, case # 12 and case # 14 had the highest antibody titers, but from a neuropathological point of view, case # 12 had little evidence of plaque removal, while case # 14 had extensive Aβ clearance. The Aβ ELISA data also show a similar pattern for case # 12 and case #14 (Table 4). Case # 15 had a relatively low antibody titer, but demonstrated a moderate amount of Aβ clearance (Table 2). Case # 21 had a low antibody titer which in the immunization context was irrelevant, since this was in an individual with PSP.

### III. Apolipoprotein E genotype

Six out of seven patients neuropathologically diagnosed as AD were ApoE genotyped. Five of them were carriers for the ε4 allele (Table 1). There was no direct correlation between the ApoE status and the total amount of GM Aβ extracted by GDFA/GHCl or those present in the Tris-soluble fractions (Table 4).

### IV. Tumor necrosis factor-α ELISA quantification

Tumor necrosis factor-α is a proinflammatory cytokine which was elevated by an average of 2.4 times in AD-immunized individuals when compared to the mean levels observed in non-immunized AD (34 pg/mg total protein, 14 pg/mg total protein, respectively; p < 0.0001, Table 4). It is noteworthy that even in the immunized AD patient with the most marked reduction in Aβ (# 14), the TNF-α level remained higher than in the non-immunized AD group (Table 4).

### V. Western blots of gray matter homogenates

**Figure 2:**
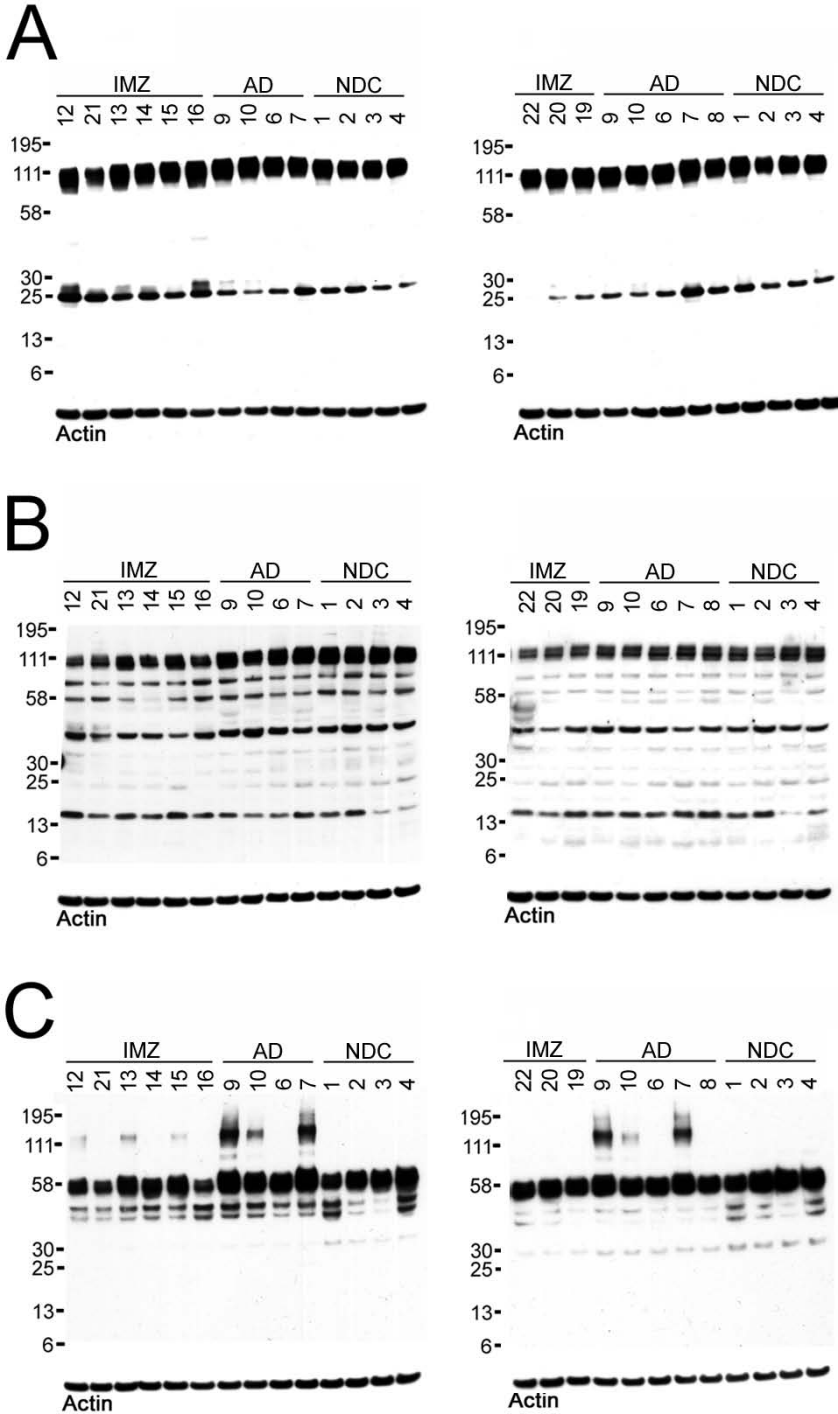
**Western blots of GM homogenates**. A total of 25 μg of protein was loaded in each lane. A) 22C11 against amino acid residues 66-81 of APP, B) CT9APP against the last nine amino acid residues of APP and C) tau HT7 against amino acid residues 159-163 of tau. For further details see the Results Section. IMZ, immunized; AD, Alzheimer's disease; NDC, non-demented control.

Cerebral cortex from the 9 immunized cases was directly homogenized in RIPA buffer and analyzed by Western blots using the 22C11, CT9APP and tau HT7 antibodies. For comparison, 4-5 AD and 4 NDC cases were also incorporated. The N-terminal-directed antibody 22C11 demonstrated no significant differences in total APP among the immunized cases as compared to non-immunized AD and NDC individuals (Figure 2A). However, there were some fluctuations in the amount of 25 kDa APP N-terminal peptide (Figure 2A). The CT9APP antibody detected the CT99/83 at ~13 kDa and a band at ~40 kDa which was present in all specimens under investigation (Figure 2B). This peptide, revealed by the CT9APP antibody, is of interest because a band of similar size is also present in PS mutations in sporadic AD and in NDC cases [38] as well as in the triple transgenic mice (3XTg) engineered by LaFerla et al. (A. Roher, unpublished observations). Further investigation is needed to clarify the nature of this putatively longer APP C-terminal peptide and its proteolytic products. There was no difference in the levels of CT99 (~13 kDa band) between the AD immunized cases and non-immunized AD cases. It is possible that the 25 and the 40 kDa bands represent complementary N-terminal and C-terminal species derived from the unmodified APP molecule. The CT9APP antibody also revealed that the amount of total APP was moderately decreased in the immunized cases when compared to non-immunized AD and NDC groups, although 22C11 did not show this trend. This antibody also demonstrated two additional APP C-terminal related bands at ~ 58 and ~ 75 kDa (Figure 2B). These observations go with the caveat that 22C11 and CT9APP can cross react with amyloid precursor-like protein (APLP). An overall comparison of the Western blot HT7 tau antibody pattern demonstrated no significant differences among AD immunized and non-immunized samples, with the exception of the AD cases # 7 and # 9 that exhibited a SDS-resistant dimeric form of tau (Figure 2C).

### VI. Column Chromatography

**Figure 3:**
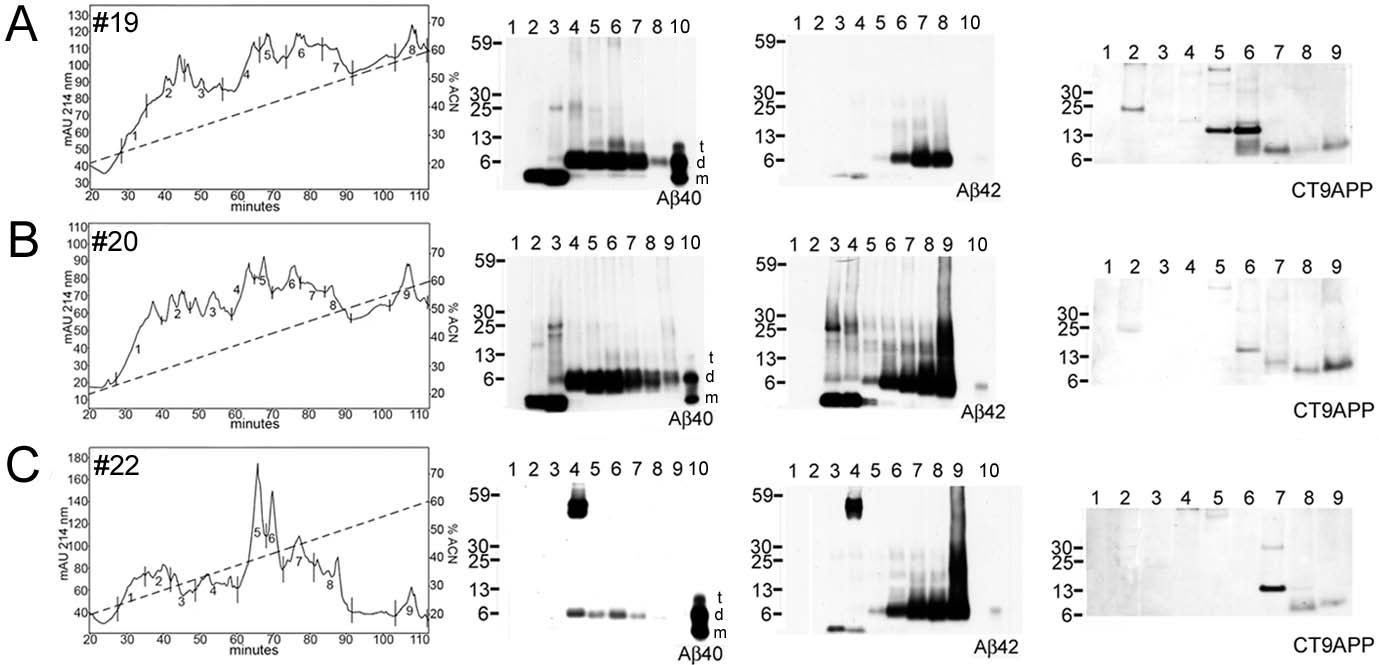
**Gray matter of UCSD immunized cases separated by HPLC (C8 reverse-phase)**. The figure shows the HPLC chromatogram fractions that were investigated by Western blotting developed with anti-Aβ40 and anti-Aβ42 and CT9APP antibodies. The diagonal-hyphenated line represents the acetonitrile gradient. Lanes 10 contain the standards Aβ40 or Aβ42 synthetic peptides. A) and B), correspond to the neuropathologically confirmed AD cases # 19 and # 20, respectively. C) corresponds to the neuropathologically confirmed hippocampal sclerosis. m, Aβ monomer; d, Aβ dimer; t, Aβ trimer.

After initial separation by FPLC, the fractions containing the Aβ peptides which were eluted between 50-62 min (equivalent to 12.5-15.5 ml of elution solvent), were separated through C8 reverse-phase HPLC (Figure 3). Biochemical characterization by Western blot was performed on 3 of the immunized cases. Case # 19 had a decreased amount of predominantly dimeric Aβ42 peptides relative to Aβ40 isoforms (Figure 3A). The AN-1792 case # 20, on the other hand, had equimolar levels of Aβ40 and Aβ42 peptides (Figure 3B). The HS case # 22 had very small amounts of Aβ40 and a more abundant complement of Aβ42-related peptides. Western blots probed with CT9APP antibody demonstrated the presence of the expected CT99 and CT83 APP C-terminal fragments at about 13 kDa in Figure 3C. In addition, they also showed a peptide band with a M_r _of ~40 kDa.

### VII. Mass spectrometry

In a previous study dealing with the remnant Aβ-related peptides left in the brains of 2 AD individuals immunized with the AN-1792, we identified a large number of peptides by matrix-assisted laser desorption/ionization-time of flight mass spectrometry (MALDI-TOF MS). The presence of these peptides was confirmed by reflectron MALDI-TOF in their monoisotopic M_r _form [28]. In the present investigation, 3 (cases # 20-22, from UCSD) out of the 9 cases under study were investigated by mass spectrometry. SELDI-TOF MS was used to identify the Aβ-related peptides using Aβ40, Aβ42 and 6E10 as capture antibodies. An assortment of Aβ-related peptides were identified with N-termini starting at 1, 2, 3-pyroglutamyl (3pE), 4, 5, 6, 11, and 17 and C-termini ending at 40, 42, 43, 46, 47, 49, 50, 61, 62, 68, 73, 82, 96 and 99 (data given in the Aβ-related amino acid sequencing, where residue 99 corresponds to residue 695 of the APP_695 _molecule, see Table 5). Dimers of Aβ 2-40 and 1-43 as well as dimeric Aβ hybrids identified as 3pE-40 + 4-40 and 3pE-40+ 2-40 were observed. As can be appreciated, there are several longer Aβ-related peptides covering amino acid sequences beyond the transmembrane domain of the APP. Our studies revealed secretase cleavage at the β-sites 1 and 11, at the α-site 17, and at putative γ-sites 40, 42, 43, 46, 47, 49, and 50. Shorter N-terminal Aβ sequences were found starting at residues 2, 3, 4, 5, and 6, probably resulting from aminopeptidase activity, as it is commonly observed in all AD cases [41]. Post-translational modifications such as cyclization of residue 3 glutamyl to pyroglutamyl [42] oxidation of Met and random formylation of Ser and Thr were also observed. The latter modification was probably an artifact of exposure of the specimens to formic acid. Although quantification of peptides by MS is difficult to achieve due to the convoluted mixture of peptides that hinders the rate of laser ionization, in AD there is a strong preponderance of dimeric Aβ-related oligomers relative to the number of monomers as suggested by our HPLC/Western blot studies (see Figure 3) and other studies performed in our laboratory that suggest dimeric Aβ as the most stable and abundant oligomeric association [43-45].

**Table 5:** SELDI-TOF peptides captured with Aβ40 and Aβ42 antibodies and 6E10

M_r _Observed	M_r _Calculated	Peptide Sequence
3371.2	3373.0	17-49 (2f)
3442.2	3444.1	17-50 (f)
3486.1	3488.1	17-50 (ox/2f)
4514.5	4514.1	1-42
4925.5	4924.7	2-47
4940.7	4942.6	1-46 (ox)
5371.4	5670.8	11-62
6178.3	6177.2	6-61 (3f)
6723.3	6724.9	11-73 (ox)
7178.2	7180.3	4-68 (3ox)
8202.0	8204.1	3pE-40 + 4-40 (4ox)
8411.7	8412.3	3pE-40 + 2-40 (ox/2f)
8450.4	8445.4	dimer 2-40 (ox)
8565.4	8563.9	5-82
9265.5	9262.4	dimer 1-43 (2ox)
9273.0	9273.7	17-99 (4ox)
9624.0	9623.1	11-96 (ox)

f, formyl; ox, oxygen; pE, pyroglutamyl

## Discussion

The outcome of active and passive Aβ immunization in APP transgenic mice models and AD patients is consistent with efficient amyloid plaque disruption, although the degree of senile core regression has been highly variable among both the treated rodents and humans. At present, more than 40 Aβ immunotherapy clinical trials involving about 10,000 subjects are being actively pursued (Alzheimer Research Forum; http://www.alzforum.org/new/detail.asp?id=2409; April 2, 2010). Interestingly, in phase II clinical trials, passive immunotherapy with Bapineuzumab showed no statistical significance in the two primary outcome measures (ADAS-cog and Disability Assessment for Dementia), although *post hoc *exploratory analyses on a smaller population of completers showed some small therapeutic effects [33,46]. However, it remains to be established whether or not anti-amyloid immunotherapy will lead to a clear improvement in cognition and quality of life enhancement for AD patients or even arrest dementia progression.

The urgent need for trustworthy biomarkers for AD is well illustrated by the proportion of potentially confounding clinical misdiagnoses discovered in the present study. Two out of the 9 individuals clinically diagnosed with AD dementia were subsequently recognized neuropathologically as PSP and HS cases. Interestingly, observations from our own brain bank at BSHRI indicate that only about 50% of the AD cases should be considered neuropathologically uncomplicated AD [36]. The remainder represents AD pathology combined with other neurodegenerative disorders, which produce severe cognitive and intellectual deterioration, such as vascular dementia, dementia with Lewy bodies, fronto-temporal dementia, Parkinson's disease, PSP, HS, or dementia without distinctive pathology. In the absence of a reliable clinical diagnosis for AD and confronted by the near or total absence of amyloid plaques in many autopsied patients who received immunotherapy, it is impossible to be certain that all these individuals actually exhibited AD plaque pathology at the onset of treatment or were at risk of developing this pathology. The introduction of imaging techniques for the detection of amyloid plaque burden such as those based on contrast-producing Aβ binding dye compounds exemplified by Pittsburgh compound-1 (^11^C-PiB-PET) will be of assistance in selecting candidates suitable for immunotherapy.

Detailed postmortem examinations of AN-1792 clinical trial participants have been undertaken by our research group and others [24-31]. A critical observation is the wide range of responses to immunotherapy reflected in the extreme variability in Aβ peptide levels among treated individuals as revealed by immunoassay studies. Assuming that the treatment commenced in patients possessing a full complement of amyloid plaques, neuropathological examination consistently demonstrated localized to extensive areas in which senile plaques apparently were disrupted. In addition, ELISA analyses suggest that in some cases the total amount of Aβ was reduced as a consequence of therapy. This inter-patient variability indicates that it will be necessary to personalize treatment regimens and titrate doses precisely to enoptimal efficacy and safety.

Our previous report [28] examined 2 AD cases immunized with the AN-1792 antigen. In one of these cases, the patient received 2 intramuscular doses of 225 μg of the immunogen. Nine months after the second immunization the patient developed non-terminal aseptic meningoencephalitis. In the second case, 3 doses of 225 μg were administered and the patient died one year after the first immunization as a result of "failure to thrive". Both individuals carried *ApoE ε3/ε4 *genotypes. In both cases, postmortem examination revealed that some areas of the brain exhibited near complete apparent amyloid plaque disruption with solubilization, but without Aβ clearance from the brain. Both vascular and diffuse amyloid deposits resisted AN-1792 disruption. The total amount of GHCl-soluble Aβ in the patient who developed meningoencephalitis was 868 μg/g wet weight compared to 186 μg/g wet weight in the second patient. The mean Aβ values in non-immunized AD (n = 31) and NDC (n = 22) were 406 μg/g wet weight and 221 μg/g wet weight, respectively [28]. However, while overall trends can be equated, these values are not directly comparable to those in the present study because the Aβ extraction technique was different and the scale of the reported means were different as well, being presented in ng/mg of total protein versus μg/g wet weight. As in the present study, total Aβ peptide levels varied widely between the two cases. Tris-soluble Aβ was 4.5 times higher and GHCl WM Aβ was 5.7 times higher in the meningoencephalitis case when compared to the second case. In the two immunized cases from the previous paper, the levels of Tris-soluble Aβ were increased over the levels in the NDC and non-immunized AD groups [28]. The same trend was repeated in the current study, where the AD immunized cases (with the exception of case # 14) had higher levels of Tris-soluble Aβ than in the means of the AD and NDC populations. In our previous study, there also was an apparent substantial increase in the amount of vascular amyloid deposits as assessed by thioflavine-S staining [28].

The fact that elimination of amyloid plaques did not alter the trajectory of decline into dementia [29] was a disappointment and suggests the disquieting possibility that amyloid plaques alone are not the direct underlying cause of dementia. Several recent studies have demonstrated that after a follow-up of two years, ^11^C-PiB uptake remains unchanged in patients with AD [47,48]. However, while amyloid deposits were stable, there was a significant decrease (~ 20%, *p *= 0.01) in regional metabolic rate for glucose [48]. Furthermore, serial ^11^C-PiB-PET and MRI in NDC, mild cognitive impairment (MCI) and AD patients demonstrated that amyloidosis alone is not sufficient to produce cognitive decline which appears to be driven by brain atrophy and neurodegeneration [49]. Interestingly, the volumes of ventricular expansion continue to increase as time progresses in NDC (1.3 ml/year), MCI (2.5 ml/year) and AD (7.7 ml/year), a manifestation of either natural aging or brain pathology-associated atrophy [49]. Amyloid plaques, although undoubtedly noxious, may represent a rescue program for the brain to manage Aβ accumulation [50]. Some studies have suggested that soluble dimeric Aβ-species represent the most toxic forms of these molecules [44,51] signifying that an exclusive focus on amyloid plaque remediation is simply too limited.

Notwithstanding disappointment in the fact that disruption of amyloid plaques neither cured dementia nor halted its progression, it is too soon to pronounce this strategy a failure. First and foremost, the hypothesis that amyloid plaques are the prime dementia-causing pathology has not yet been tested rigorously. Biochemical dissection has revealed that amyloid plaques are more than accumulated Aβ, but actually represent complex multi-molecular assemblages [52,53]. Postmortem examinations have revealed that some amyloid plaques are not reversed completely by immunotherapy [28] and remnants, dubbed "collapsed" plaques [25] or "moth-eaten" plaques [27] composed of insoluble molecules persist. It is possible that despite the impressive morphological effects of immunotherapy, "amyloid plaque skeletal remnants" harbor toxic moieties and continue to exert a deleterious legacy effect on dementia development. In addition, the Aβ spectrum composing plaque deposits may be sufficiently diverse in structure to thwart complete disruption by the immunotherapeutic agents employed to date. The humanized monoclonal antibodies that recognize the Aβ molecule N-terminus [46] will fail to recognize the terminally-truncated Aβ species demonstrated to be prevalent in human senile plaques [41]. Furthermore, diffuse plaques lack surrounding reactive microglia and fall short of eliciting an inflammatory response [54,55]. This phenomenon may be due to the absence of the Aβ HHQK domain that binds to the glycosaminoglycans on the surface of microglia [13,14]. Furthermore, diffuse plaques that are mainly composed of P3 (Aβ residues 17-42 [56]), may elude disruption by immunotherapy directed to the N-terminal domain of Aβ. The α-secretase cleavage does not generate amyloidogenic peptides and therefore it has been considered a favorable therapeutic pathway. However, there is evidence supporting the contention the P3 activates JNK and caspase-8 resulting in neuronal apoptosis [57]. In addition to amyloid and diffuse plaque deposits, AD patients harbor NFT and these lesions have persisted in patients who have received anti-Aβ immunotherapy [24,25,27,28,31,35]. Because several distinct classes of pathological and biochemical lesions typically co-exist in AD patients, a complete cure for dementia may simply need to address more than amyloid plaques.

The broad-scale, chronic use of anti-amyloid plaque immunotherapy is complicated by the fact that the fundamental function(s) of the evolutionarily-conserved Aβ molecules remain unknown. One possibility is that amyloid deposits perform a vascular damage rescue function by forming a patch wherever the blood brain barrier (BBB) is breached [17]. Studies in transgenic mice and humans reveal that Aβ immunotherapy exerts powerful, sometimes deleterious effects on vascular integrity and function [30,31,58-60]. In addition, direct clinical experience has confirmed that individuals harboring *ApoEε4 *genes are more likely to suffer severe adverse effects from amyloid immunotherapy. While it is unclear whether these responses are a cause or consequence of vascular pathology, they unfortunately do reveal that AN-1792 is contraindicated outright or must be applied with caution in the patient subpopulation known to be most at risk for AD development.

The enhanced production of TNF-α in the immunized AD cases revealed an ongoing inflammatory reaction in part generated by antigen-antibody interactions. Opsonization of Aβ and subsequent uptake by microglia through the Fc receptors stimulates the secretion of molecules such as interleukin (IL)-1, IL-6, IL-10, TNF-α and macrophage colony stimulating factor that promote neuroinflammation and opening of the BBB. These paradoxical reactions need to be considered in the design of effective immunotherapies [61]. In addition, TNF-α suppresses Aβ degradation by reducing the expression of insulin degrading enzyme [62].

Despite a decade of promising developments in amyloid plaque mitigation, whether the amyloid hypothesis represents the ultimate mechanistic explanation for sporadic AD pathology remains undetermined. Regardless of the role of amyloid plaques as the leading cause for dementia, the existing efforts to combat these lesions need further development. In addition to examining the pathophysiology underlying the remarkable amyloid plaque disruption in the brain following immunotherapy, an account of the role of peripheral pools in increasing levels of circulating Aβ requires a full mechanistic explanation.

## Conclusions

In summary, our results revealed a wide variation in the overall proportions of Aβ40 and Aβ42 peptides among the immunized individuals, with a general predominance of SDS-stable dimeric forms over monomeric ones. In addition, SELDI-TOF MS demonstrated an array of Aβ-related peptides, mainly extending towards the C-terminal domain of APP that, in part, may result from the strong extraction conditions that we utilized, capable of totally dispersing membrane structures. Pro-inflammatory TNF-α levels were significantly increased in the GM of immunized AD cases compared to the NDC and non-immunized AD groups. Amyloid-β immunization resulted in amyloid plaque disruption and clearance to widely divergent extents, with levels ranging from below the limit of detection to values exceeding those seen in non-immunized AD cases in ELISA. These idiosyncratic responses, in conjunction with the wide range of biological and pathological variation that characterize aging and disease conditions, will make it difficult to interpret data derived from therapeutic investigations. Moreover, this variation enormously complicates recognition of suitable biomarkers for AD. Our data suggest that the therapeutic outcome will depend on the quality and the quantity of administered immunogens and the patient's immunological responses with the ApoE phenotype appearing to modulate the effectiveness of plaque and vascular amyloid removal. In attempting to deploy Aβ antibodies as AD therapeutic tools, the full range of physicochemical properties of the Aβ peptides in humans should be considered. Although in some cases the removal of amyloid plaques by AN-1792 appeared to be impressive, there were no proportionate alterations in the clinical progression of Alzheimer's disease.

## List of Abbreviations

^11^C-PiB: Pittsburgh compound; Aβ: amyloid-beta; AD: Alzheimer's disease; ApoE: apolipoprotein E; APP: amyloid-beta precursor protein; BBB: blood brain barrier; BSHRI: Banner Sun Health Research Institute; CAA: cerebral amyloid angiopathy; CERAD: Consortium to Establish a Registry for Alzheimer's disease; CHCA: α-cyano-4-hydroxycinnamic acid; F: female; f: formyl; FLA: frontal lobe atrophy; FPLC: fast protein liquid chromatography; GDFA: glass distilled formic acid; GHCl: guanidine hydrochloride; GM: gray matter; HPLC: high performance liquid chromatography; HS: hippocampal sclerosis; IL: interleukin; Imm: immunization; M: male; MALDI-TOF: matrix-assisted laser desorption/ionization-time of flight; MCI: mild cognitive impairment; MMSE: mini mental state examination; MS: mass spectrometry; n/a: not available; NDC: non-demented control; NFT: neurofibrillary tangles; Non-ADD: non-Alzheimer's disease dementias; NP: neuritic plaque; ox: oxygen; pE: pyroglutamyl; PIC: protease inhibitor cocktail; PS: presenilin; PSP: progressive supranuclear palsy; TFA: trifluoroacetic acid; Tg: transgenic; TNF-α: tumor necrosis factor-alpha; USCD: University of California San Diego; USSM: University of Southampton School of Medicine; PBS: phosphate buffered saline; RT: room temperature; SELDI-TOF: surface enhanced laser desorption/ionization-time of flight; WM: white matter; WMR: white matter rarefaction

## Declaration of Competing interests

JARN is a consultant/advisor relating to Alzheimer immunization programs: Elan Pharmaceuticals, GSK, Novartis, Roche, Janssen Alzheimer Immunotherapy Research and Development. MNS receives grant support (clinical trials) from BMS, Avid, GE, Bayer, Baxter, Wyeth, Janssen, Lilly and Medivation. MNS is also on the Consultant/advisory board for Janssen/Pfizer, Amerisciences, Eisai and GSK, and receives royalties from Amerisciences and Wiley. TGB receives funding from AVID-Bayer GE Radiopharmaceuticals. The remaining authors have no competing interests.

## Authors' contributions

MNS and TGB provided the clinical and neuropathological assessments of the AD and NDC individuals. JAN and EM provided clinical and neuropathological data of AN-1792 cases. WMK, RLP, DCL, CLM and IDD were involved in the collection and analysis of the data. AER, TAK and EMC were involved in the design of experiments and final production of the manuscript. All the authors participated in revising and editing of the manuscript.

## References

[B1] Brookmeyer R Johnson E Ziegler-Graham K Arrighi HM Forecasting the global burden of Alzheimer's disease Alzheimers Dement 2007 3 186 191 10.1016/j.jalz.2007.04.381 19595937

[B2] 2010 Alzheimer's disease facts and figures Alzheimers Dement 2010 6 158 194 10.1016/j.jalz.2010.01.009 20298981

[B3] Glenner GG Wong CW Alzheimer's disease: initial report of the purification and characterization of a novel cerebrovascular amyloid protein Biochem Biophys Res Commun 1984 120 885 890 10.1016/S0006-291X(84)80190-4 6375662

[B4] Masters CL Simms G Weinman NA Multhaup G McDonald BL Beyreuther K Amyloid plaque core protein in Alzheimer disease and Down syndrome Proc Natl Acad Sci USA 1985 82 4245 4249 10.1073/pnas.82.12.4245 3159021 PMC397973

[B5] Hardy JA Higgins GA Alzheimer's disease: the amyloid cascade hypothesis Science 1992 256 184 185 10.1126/science.1566067 1566067

[B6] Selkoe DJ Alzheimer's disease: genes, proteins, and therapy Physiol Rev 2001 81 741 766 11274343 10.1152/physrev.2001.81.2.741

[B7] Joseph J Shukitt-Hale B Denisova NA Martin A Perry G Smith MA Copernicus revisited: amyloid beta in Alzheimer's disease Neurobiol Aging 2001 22 131 146 10.1016/S0197-4580(00)00211-6 11164287

[B8] Obrenovich ME Joseph JA Atwood CS Perry G Smith MA Amyloid-beta: a (life) preserver for the brain Neurobiol Aging 2002 23 1097 1099 10.1016/S0197-4580(02)00038-6 12470809

[B9] Lopez-Toledano MA Shelanski ML Neurogenic effect of beta-amyloid peptide in the development of neural stem cells J Neurosci 2004 24 5439 5444 10.1523/JNEUROSCI.0974-04.2004 15190117 PMC6729298

[B10] Robinson SR Bishop GM Abeta as a bioflocculant: implications for the amyloid hypothesis of Alzheimer's disease Neurobiol Aging 2002 23 1051 1072 10.1016/S0197-4580(01)00342-6 12470802

[B11] Bishop GM Robinson SR The amyloid hypothesis: let sleeping dogmas lie? Neurobiol Aging 2002 23 1101 1105 10.1016/S0197-4580(02)00050-7 12470810

[B12] Plant LD Boyle JP Smith IF Peers C Pearson HA The production of amyloid beta peptide is a critical requirement for the viability of central neurons J Neurosci 2003 23 5531 5535 12843253 10.1523/JNEUROSCI.23-13-05531.2003 PMC6741264

[B13] Giulian D Haverkamp LJ Yu JH Karshin W Tom D Li J Specific domains of beta-amyloid from Alzheimer plaque elicit neuron killing in human microglia J Neurosci 1996 16 6021 6037 8815885 10.1523/JNEUROSCI.16-19-06021.1996 PMC6579176

[B14] Giulian D Haverkamp LJ Yu J Karshin W Tom D Li J The HHQK domain of beta-amyloid provides a structural basis for the immunopathology of Alzheimer's disease J Biol Chem 1998 273 29719 29726 10.1074/jbc.273.45.29719 9792685

[B15] Thomas T Thomas G McLendon C Sutton T Mullan M beta-Amyloid-mediated vasoactivity and vascular endothelial damage Nature 1996 380 168 171 10.1038/380168a0 8600393

[B16] Paris D Townsend K Quadros A Humphrey J Sun J Brem S Inhibition of angiogenesis by Abeta peptides Angiogenesis 2004 7 75 85 10.1023/B:AGEN.0000037335.17717.bf 15302999

[B17] Roher AE Lowenson JD Clarke S Woods AS Cotter RJ Gowing E beta-Amyloid-(1-42) is a major component of cerebrovascular amyloid deposits: implications for the pathology of Alzheimer disease Proc Natl Acad Sci USA 1993 90 10836 10840 10.1073/pnas.90.22.10836 8248178 PMC47873

[B18] Atwood CS Bowen RL Smith MA Perry G Cerebrovascular requirement for sealant, anti-coagulant and remodeling molecules that allow for the maintenance of vascular integrity and blood supply Brain Res Brain Res Rev 2003 43 164 178 10.1016/S0165-0173(03)00206-6 14499467

[B19] Roskam S Neff F Schwarting R Bacher M Dodel R APP transgenic mice: the effect of active and passive immunotherapy in cognitive tasks Neurosci Biobehav Rev 2010 34 487 499 10.1016/j.neubiorev.2009.10.006 19857518

[B20] Loerch PM Lu T Dakin KA Vann JM Isaacs A Geula C Evolution of the aging brain transcriptome and synaptic regulation PLoS One 2008 3 e3329 10.1371/journal.pone.0003329 18830410 PMC2553198

[B21] Roher AE Kokjohn TA Appraisal of AbetaPP Transgenic Mice as Models for Alzheimer's Disease Amyloid Cascade Curr Med Chem Immun Endo & Metab Agents 2003 3 85 90

[B22] Kokjohn TA Roher AE Amyloid precursor protein transgenic mouse models and Alzheimer's disease: understanding the paradigms, limitations, and contributions Alzheimers Dement 2009 5 340 347 10.1016/j.jalz.2009.03.002 19560104 PMC2704491

[B23] Kokjohn TA Roher AE Antibody responses, amyloid-beta peptide remnants and clinical effects of AN-1792 immunization in patients with AD in an interrupted trial CNS Neurol Disord Drug Targets 2009 8 88 97 10.2174/187152709787847315 19355930 PMC2742220

[B24] Nicoll JA Wilkinson D Holmes C Steart P Markham H Weller RO Neuropathology of human Alzheimer disease after immunization with amyloid-beta peptide: a case report Nat Med 2003 9 448 452 10.1038/nm840 12640446

[B25] Ferrer I Boada RM Sanchez Guerra ML Rey MJ Costa-Jussa F Neuropathology and pathogenesis of encephalitis following amyloid-beta immunization in Alzheimer's disease Brain Pathol 2004 14 11 20 10.1111/j.1750-3639.2004.tb00493.x 14997933 PMC8095815

[B26] Masliah E Hansen L Adame A Crews L Bard F Lee C Abeta vaccination effects on plaque pathology in the absence of encephalitis in Alzheimer disease Neurology 2005 64 129 131 15642916 10.1212/01.WNL.0000148590.39911.DF

[B27] Nicoll JA Barton E Boche D Neal JW Ferrer I Thompson P Abeta species removal after abeta42 immunization J Neuropathol Exp Neurol 2006 65 1040 1048 10.1097/01.jnen.0000240466.10758.ce 17086100

[B28] Patton RL Kalback WM Esh CL Kokjohn TA Van Vickle GD Luehrs DC Amyloid-beta peptide remnants in AN-1792-immunized Alzheimer's disease patients: a biochemical analysis Am J Pathol 2006 169 1048 1063 10.2353/ajpath.2006.060269 16936277 PMC1698828

[B29] Holmes C Boche D Wilkinson D Yadegarfar G Hopkins V Bayer A Long-term effects of Abeta42 immunisation in Alzheimer's disease: follow-up of a randomised, placebo-controlled phase I trial Lancet 2008 372 216 223 10.1016/S0140-6736(08)61075-2 18640458

[B30] Boche D Zotova E Weller RO Love S Neal JW Pickering RM Consequence of Abeta immunization on the vasculature of human Alzheimer's disease brain Brain 2008 131 3299 3310 10.1093/brain/awn261 18953056

[B31] Uro-Coste E Russano de PG Guilbeau-Frugier C Sastre N Ousset PJ da Silva NA Cerebral amyloid angiopathy and microhemorrhages after amyloid beta vaccination: case report and brief review Clin Neuropathol 2010 29 209 216 20569670 10.5414/npp29209

[B32] Orgogozo JM Gilman S Dartigues JF Laurent B Puel M Kirby LC Subacute meningoencephalitis in a subset of patients with AD after Abeta42 immunization Neurology 2003 61 46 54 12847155 10.1212/01.wnl.0000073623.84147.a8

[B33] Salloway S Sperling R Gilman S Fox NC Blennow K Raskind M A phase 2 multiple ascending dose trial of bapineuzumab in mild to moderate Alzheimer disease Neurology 2009 73 2061 2070 10.1212/WNL.0b013e3181c67808 19923550 PMC2790221

[B34] Serrano-Pozo A William CM Ferrer I Uro-Coste E Delisle MB Maurage CA Beneficial effect of human anti-amyloid-beta active immunization on neurite morphology and tau pathology Brain 2010 133 1312 1327 10.1093/brain/awq056 20360050 PMC2859150

[B35] Boche D Donald J Love S Harris S Neal JW Holmes C Reduction of aggregated Tau in neuronal processes but not in the cell bodies after Abeta42 immunisation in Alzheimer's disease Acta Neuropathol 2010 120 13 20 10.1007/s00401-010-0705-y 20532897

[B36] Beach TG Sue LI Walker DG Roher AE Lue L Vedders L The Sun Health Research Institute Brain Donation Program: description and experience, 1987-2007 Cell Tissue Bank 2008 9 229 245 10.1007/s10561-008-9067-2 18347928 PMC2493521

[B37] Mulugeta E Molina-Holgado F Elliott MS Hortobagyi T Perry R Kalaria RN Inflammatory mediators in the frontal lobe of patients with mixed and vascular dementia Dement Geriatr Cogn Disord 2008 25 278 286 10.1159/000118633 18303264

[B38] Maarouf CL Daugs ID Spina S Vidal R Kokjohn TA Patton RL Histopathological and molecular heterogeneity among individuals with dementia associated with Presenilin mutations Mol Neurodegener 2008 3 20 10.1186/1750-1326-3-20 19021905 PMC2600784

[B39] Esh C Patton L Kalback W Kokjohn TA Lopez J Brune D Altered APP processing in PDAPP (Val717 --> Phe) transgenic mice yields extended-length Abeta peptides Biochemistry 2005 44 13807 13819 10.1021/bi051213+ 16229470

[B40] Dean RB Dixon WJ Simplified statistics for small numbers of observations Anal Chem 1951 23 636 638 10.1021/ac60052a025

[B41] Roher AE Lowenson JD Clarke S Wolkow C Wang R Cotter RJ Structural alterations in the peptide backbone of beta-amyloid core protein may account for its deposition and stability in Alzheimer's disease J Biol Chem 1993 268 3072 3083 8428986

[B42] Kuo YM Emmerling MR Woods AS Cotter RJ Roher AE Isolation, chemical characterization, and quantitation of A beta 3-pyroglutamyl peptide from neuritic plaques and vascular amyloid deposits Biochem Biophys Res Commun 1997 237 188 191 10.1006/bbrc.1997.7083 9266855

[B43] Kuo YM Emmerling MR Vigo-Pelfrey C Kasunic TC Kirkpatrick JB Murdoch GH Water-soluble Abeta (N-40, N-42) oligomers in normal and Alzheimer disease brains J Biol Chem 1996 271 4077 4081 10.1074/jbc.271.8.4077 8626743

[B44] Roher AE Chaney MO Kuo YM Webster SD Stine WB Haverkamp LJ Morphology and toxicity of Abeta-(1-42) dimer derived from neuritic and vascular amyloid deposits of Alzheimer's disease J Biol Chem 1996 271 20631 20635 10.1074/jbc.271.34.20631 8702810

[B45] Kuo YM Webster S Emmerling MR De LN Roher AE Irreversible dimerization/tetramerization and post-translational modifications inhibit proteolytic degradation of A beta peptides of Alzheimer's disease Biochim Biophys Acta 1998 1406 291 298 9630681 10.1016/s0925-4439(98)00014-3

[B46] Kerchner GA Boxer AL Bapineuzumab Expert Opin Biol Ther 2010 10 1121 1130 10.1517/14712598.2010.493872 20497044 PMC3000430

[B47] Scheinin NM Aalto S Koikkalainen J Lotjonen J Karrasch M Kemppainen N Follow-up of [11C]PIB uptake and brain volume in patients with Alzheimer disease and controls Neurology 2009 73 1186 1192 10.1212/WNL.0b013e3181bacf1b 19726751

[B48] Engler H Forsberg A Almkvist O Blomquist G Larsson E Savitcheva I Two-year follow-up of amyloid deposition in patients with Alzheimer's disease Brain 2006 129 2856 2866 10.1093/brain/awl178 16854944

[B49] Jack CR Jr Lowe VJ Weigand SD Wiste HJ Senjem ML Knopman DS Serial PIB and MRI in normal, mild cognitive impairment and Alzheimer's disease: implications for sequence of pathological events in Alzheimer's disease Brain 2009 132 1355 1365 10.1093/brain/awp062 19339253 PMC2677798

[B50] Heininger K A unifying hypothesis of Alzheimer's disease. IV. Causation and sequence of events Rev Neurosci 2000 11 Spec No 213 328 11065271 10.1515/revneuro.2000.11.s1.213

[B51] Shankar GM Li S Mehta TH Garcia-Munoz A Shepardson NE Smith I Amyloid-beta protein dimers isolated directly from Alzheimer's brains impair synaptic plasticity and memory Nat Med 2008 14 837 842 10.1038/nm1782 18568035 PMC2772133

[B52] Liao L Cheng D Wang J Duong DM Losik TG Gearing M Proteomic characterization of postmortem amyloid plaques isolated by laser capture microdissection J Biol Chem 2004 279 37061 37068 10.1074/jbc.M403672200 15220353

[B53] Roher AE Palmer KC Yurewicz EC Ball MJ Greenberg BD Morphological and biochemical analyses of amyloid plaque core proteins purified from Alzheimer disease brain tissue J Neurochem 1993 61 1916 1926 10.1111/j.1471-4159.1993.tb09834.x 8229002

[B54] Wisniewski HM Wegiel J The neuropathology of Alzheimer's disease Neuroimaging Clin N Am 1995 5 45 57 7743084

[B55] Mrak RE Neuropathology and the neuroinflammation idea J Alzheimers Dis 2009 18 473 481 19584454 10.3233/JAD-2009-1158

[B56] Gowing E Roher AE Woods AS Cotter RJ Chaney M Little SP Chemical characterization of A beta 17-42 peptide, a component of diffuse amyloid deposits of Alzheimer disease J Biol Chem 1994 269 10987 10990 8157623

[B57] Wei W Norton DD Wang X Kusiak JW Abeta 17-42 in Alzheimer's disease activates JNK and caspase-8 leading to neuronal apoptosis Brain 2002 125 2036 2043 10.1093/brain/awf205 12183349

[B58] Racke MM Boone LI Hepburn DL Parsadainian M Bryan MT Ness DK Exacerbation of cerebral amyloid angiopathy-associated microhemorrhage in amyloid precursor protein transgenic mice by immunotherapy is dependent on antibody recognition of deposited forms of amyloid beta J Neurosci 2005 25 629 636 10.1523/JNEUROSCI.4337-04.2005 15659599 PMC6725332

[B59] Wilcock DM Colton CA Immunotherapy, vascular pathology, and microhemorrhages in transgenic mice CNS Neurol Disord Drug Targets 2009 8 50 64 10.2174/187152709787601858 19275636 PMC2659468

[B60] Burbach GJ Vlachos A Ghebremedhin E Del Turco D Coomaraswamy J Staufenbiel M Vessel ultrastructure in APP23 transgenic mice after passive anti-Abeta immunotherapy and subsequent intracerebral hemorrhage Neurobiol Aging 2007 28 202 212 10.1016/j.neurobiolaging.2005.12.003 16427722

[B61] Lue LF Walker DG Modeling Alzheimer's disease immune therapy mechanisms: interactions of human postmortem microglia with antibody-opsonized amyloid beta peptide J Neurosci Res 2002 70 599 610 10.1002/jnr.10422 12404514

[B62] Yamamoto M Kiyota T Walsh SM Liu J Kipnis J Ikezu T Cytokine-mediated inhibition of fibrillar amyloid-beta peptide degradation by human mononuclear phagocytes J Immunol 2008 181 3877 3886 18768842 10.4049/jimmunol.181.6.3877 PMC2603577

[B63] Blessed G Tomlinson BE Roth M The association between quantitative measures of dementia and of senile change in the cerebral grey matter of elderly subjects Br J Psychiatry 1968 114 797 811 10.1192/bjp.114.512.797 5662937

